# Tau-focused therapy and tau transmission: Implications for Alzheimer’s Disease and related tauopathies

**DOI:** 10.1186/1750-1326-8-S1-O37

**Published:** 2013-09-13

**Authors:** John Q Trojanowski

**Affiliations:** 1 University of Pennsylvania, Philadelphia, USA

## 

Tau pathology is characteristic of Alzheimer’s disease (AD) and related tauopathies including Parkinson’s disease (PD) wherein a significant percentage of PD patients who develop dementia harbor AD-like tau pathology. Tau pathology is characterized by insoluble filamentous aggregates of hyperphosphorylated tau in CNS neurons known as neurofibrillary tangles (NFTs) and neuropil threads (NTs). NFTs and NTs are implicated in mechanisms of neurodegeneration through gains of toxic functions or losses of normal tau functions. Hyperphosphorylation and decreased solubility of tau leads to a loss of the ability of tau to bind to and stabilize microtubules (MTs) which impairs axonal transport and results in neuronal dysfunction and degeneration. This presentation describes the use of MT stabilizing drugs to counteract the loss of tau function and prevent/ameliorate tau mediated neurodegeneration in a transgenic (Tg) mouse model of an AD-like neurodegenerative tauopathy. Briefly, proof of concept prevention and intervention studies of the brain-penetrant MT-stabilizing agent epothilone D (EpoD) are described in young (prevention) and aged (intervention) P301S mutant tau Tg mice (PS 19 mice) with little (young PS 19 mice) or abundant tau pathology and behavioral impairments (aged PS19 mice). In the prevention and intervention studies, EpoD treatment reduced axonal dystrophy and increased axonal MT density which led to improved neuronal integrity and cognitive performance. EpoD treatment also improved fast axonal transport in the aged PS19 Tg mice. Unexpectedly, aged PS19 mice treated with EpoD showed a marked reduction in forebrain tau pathology and increased hippocampal neuronal integrity. There were no dose-limiting side effects in the EpoD treated young and aged PS19 mice. These studies support the view that EpoD and other brain-penetrant MT-stabilizing drugs offset the loss of tau function in neurodegenerative tauopathies and therefore are potential therapeutics for the treatment of AD and related tauopathies. Finally, a new seeded transmission model of tauopathy is presented using injections of preformed tau fibrils into the brains of PS19 mice which presents new opportunities to assess the efficacy of MT stabilizers and other potential passive immunization therapies for AD and related tauopathies.

Supported by grants from the NINDS/NIA.

